# Workplace psychological violence among intern nursing students and its relationship with professional commitment: the moderating role of gender

**DOI:** 10.3389/fpubh.2026.1778729

**Published:** 2026-03-27

**Authors:** YaRu Zhong, YaQin Li, HongWei Yang, ChunHuan Sun, Man Li, JingFang Zhao

**Affiliations:** 1 Department of Gastroenterology, Shandong Provincial Hospital Affiliated to Shandong First Medical University, Jinan, China; 2 Department of Oral and Maxillofacial Surgery, Shandong Provincial Hospital Affiliated to Shandong First Medical University, Jinan, China

**Keywords:** nursing intern, nursing students, professional commitment, psychological violence, workplace violence

## Abstract

**Background:**

The quality of clinical nursing services and the retention of nursing talent depend heavily on intern nurses’ dedication to their careers. A clinical internship is an essential phase in the development of nurses’ commitment to their careers. As a prevalent detrimental element in the clinical setting, workplace psychological violence is seen as a significant factor influencing intern nurses’ degree of professional dedication. Although gender variations may cause nurses to perceive psychological violence differently and use different coping mechanisms, it is unclear how these differences moderate the association between psychological violence and professional commitment.

**Objective:**

Examine the relationship between intern nursing students’ professional commitment and psychological violence at work, analyzing how gender influences this relationship.

**Method:**

A total of 1,095 intern nurses from 14 medical institutions in Shandong Province were included as study subjects by using the convenience sampling method. The survey was conducted through an electronic questionnaire. The research tools included the Nursing Professional Commitment Scale and the Psychological Violence Behavioiur scale for nursing students (WPVB). Pearson correlation analysis was used to explore the relationship between workplace psychological violence and professional commitment among nursing staff. Stratified multiple linear regression models and mixed regression models including the interaction effect of WPVB × gender (and covariates) were used to test the moderating role of gender on the relationship between them. A two-sided *p* value < 0.05 was considered statistically significant.

**Result:**

The Pearson correlation analysis showed that workplace psychological violence (WPVB) was negatively correlated with professional commitment among intern nurses. Hierarchical multiple linear regression analysis indicated that the negative association between WPVB and professional commitment was statistically significant only among female intern nurses, whereas no significant correlation was found in the male group. However, mixed linear regression models incorporating the WPVB × gender interaction term and demographic and sociological covariates revealed that the interaction term was not statistically significant across all stepwise regression models (Model 1: Estimate = −0.022, *p* = 0.7424; Model 2: Estimate = −0.019, *p* = 0.7742; Model 3: Estimate = −0.040, *p* = 0.53898), indicating that gender did not significantly moderate the relationship between WPVB and professional commitment among intern nurses.

**Conclusion:**

Workplace psychological violence (WPVB) is a prevalent issue among nursing interns, negatively impacting their professional commitment. Although female interns report higher incidences of WPVB compared to male counterparts, gender does not significantly mediate the relationship between WPVB and professional commitment. This indicates that targeted intervention strategies should be developed by nursing educators and managers to mitigate workplace psychological violence and bolster the professional commitment of nursing interns.

## 1 Introduction

Professional commitment, defined as “an individual’s attitude and identification with their profession” or “the motivation for choosing a profession and loyalty to it” (1), encompasses three core factors: belief in professional goals and values, willingness to exert effort to understand these values, and determination to remain in the profession (2). This concept is a critical component of nursing education and practice, being directly linked to intern nurse retention rates and career development, and closely correlated with the delivery of high-quality nursing services (3). Studies indicate that professional commitment during the internship phase serves as a significant predictor of nursing students’ decision to pursue a nursing career post-graduation (4); specifically, higher levels of professional commitment among students correlate with an increased likelihood of entering the nursing profession after graduation (5, 6). Furthermore, strong professional commitment motivates nursing students to engage proactively in continuous learning, stay current with healthcare advancements, and advance their professional growth (7). Additionally, professional commitment can mitigate the adverse effects of high-pressure work environments (8), as individuals with high levels of this commitment are more inclined to utilize available resources to overcome challenges, enabling them to approach clinical practice with a positive mindset (9), enhance their dedication to delivering high-quality care, and ultimately elevate the overall standard of nursing services (2).

The clinical internship is a pivotal phase in nursing education. It not only plays an instrumental role in cultivating professional attitudes, fostering emotional engagement, and solidifying professional identity, but it also represents a critical period for the development of nursing students’ professional commitment (10, 11). The level of professional commitment exhibited by nursing students during their clinical internships is significantly influenced by their environment (1, 12). A supportive professional environment can enhance the degree of professional commitment among nursing students, whereas adverse environmental factors may impede the growth of their professional commitment. One such negative factor that frequently occurs in the clinical environment is psychological violence, which has emerged as a significant issue in global public health. Nursing students, due to their limited clinical knowledge and skills and their lack of ability to manage clinical emergencies, are particularly susceptible to psychological violence (12).

Psychological violence, characterized by intentional actions that harm an individual’s physical, mental, or social well-being, manifests primarily as verbal abuse, threats, insults, and harassment (13). Numerous epidemiological studies have documented the pervasive exposure of nursing interns to psychological violence globally: a UK-based study revealed that approximately 42.2% of students experienced bullying/harassment in the preceding year (14), while in Turkey, nearly 91.6% faced verbal violence (15). In Hong Kong, Cheung et al. found that 30.6% of nursing interns reported experiencing verbal threats (16). Similarly, a survey of 195 nursing interns in Chinese tertiary general hospitals by Qu Dan et al. indicated a 60% incidence rate of psychological violence (17). Exposure to workplace psychological violence is associated with adverse emotional outcomes such as anxiety, depression, and fear, which diminish quality of life (18, 19); it also erodes trust and collaboration between nurses and patients, compromising the quality and safety of patient care (20); furthermore, it may foster intentions to quit among nursing interns during their clinical placements (21–23), thereby exacerbating the shortage of nursing personnel.

In recent years, researchers have begun to pay attention to the impact of psychological violence on career commitment among nursing students. Studies have shown that exposure to workplace psychological violence can weaken career commitment in nursing students (24, 25). However, despite these insights, the potential moderating role of gender in this relationship remains underexplored and warrants further investigation. Gender differences may lead to distinct coping patterns among nursing students when confronted with psychological violence. Social role theory suggests that men and women evaluate and respond to stressful events differently based on their respective social roles (26). The behavioral differences between men and women originate from their divergent social roles, which result in different divisions of labor and consequently shape distinct gender role expectations and skill sets (27). In social interactions, females tend to be more relationally oriented and are more susceptible to negative self-concepts and social situational cognition factors (28). Workplace psychological violence is likely to thwart the fulfillment of individuals’ fundamental social needs, making it difficult for them to find meaning and a sense of self-worth in their work, potentially leading to diminished career commitment. This implies that gender may moderate an individual’s perception and response to psychological violence, thereby influencing the strength or direction of the association between psychological violence and career commitment. Against this backdrop, the present study focuses on a cohort of intern nursing students to explore the association between workplace psychological violence and career commitment, specifically testing for the moderating effect of gender.

## 2 Methods

### 2.1 Design and participants

This study employed a cross-sectional design. Between April and July 2021, third- and fourth-year nursing students currently engaged in clinical practice at 14 medical colleges in Shandong Province were conveniently sampled as research participants. The inclusion criteria specified that participants must be enrolled in a nursing major, actively participating in clinical practice for a duration of at least six months, and have signed a written informed consent form to participate in the study, the exclusion criteria encompassed individuals who were unable to partake in the survey due to reasons such as sick leave or personal leave during the investigation period, as well as those nursing students who were concurrently involved in other research studies.

### 2.2 Instrument tools

The general information questionnaire was developed based on a literature review and the research objectives. It comprised 11 items, including gender, age, hospital level, education level, only-child status, family residence, class cadre status, scholarship receipt, physical activity, school position status, and clinical internship period.

Workplace Psychological Violence Scale: This scale was developed by Yildirim et al. (13) and adapted and revised by Xu Meizi et al. (29). It consists of 32 items covering four dimensions: being isolated at work (Individual’s isolation from work) (10 items), attack on professional status (Attack on professional status) (9 items), personality attack (Attack on personality) (7 items), and direct negative behaviors (Direct negative behaviors) (6 items). A Likert 6-point scale is used (0 = never occurred, 5 = always occurred). The higher the score, the more frequently psychological violence has been experienced. In this study, the item-level content validity index was 0.78–0.86, the Cronbach’s *α* coefficient of the Workplace Psychological Violence Behavior Scale was 0.942 for the overall sample, 0.940 for females, and 0.959 for males.

Nursing Professional Commitment Scale: this scale was developed by Taiwanese scholar Lu Guiyun (30) and later revised (31) to investigate the level of nursing professional commitment among nursing interns. It comprises 34 items across four dimensions: Professional effort willingness (16 items), Professional career involvement (9 items), Positive professional evaluation (5 items), and Professional value identification (4 items). The scale employs a Likert 4-point scale (1 = extremely uncertain, 4 = very certain), with higher total scores indicating greater commitment levels. In this study, Cronbach’s *α* coefficient for the Nursing Professional Commitment Scale was 0.878 for the total sample, 0.882 for female participants, and 0.856 for male participants.

### 2.3 Data collection

The questionnaire was developed using the online platform Questionnaire Star, and the survey was conducted anonymously. The investigators consisted of teachers from 14 medical colleges. Prior to participation, these teachers received training from the researchers; only those who passed this training subsequently participated in the survey. During the data collection process, the investigators explained the purpose and significance of the study and provided instructions on how to complete the questionnaire. A standardized guideline was utilized to address any issues encountered by trainee nurses during the survey. In terms of ethics, all participants signed an informed consent form before participating in the study.

### 2.4 Data analysis

Data analysis was performed using SPSS version 27.0 and AMOS version 24.0. Continuous variables were expressed as mean (standard deviation). The independent sample t-test was used to analyze the differences in psychological violence and professional commitment scores between male and female nursing students; Pearson correlation analysis was used to explore the correlation between workplace psychological violence (WPVB) and professional commitment among nursing trainees; Stratified multiple linear regression model was used to evaluate the differences of workplace psychological violence (WPVB) and professional commitment between different genders. Two-sided *p* < 0.05 was considered statistically significant.

### 2.5 Ethical considerations

This study was approved by the Ethics Committee of Provincial Hospital Affiliated to Shandong First Medical University (No.2022–561). In accordance with the provisions of the Declaration of Helsinki, the personal data of respondents were strictly confidential and their privacy was protected.

## 3 Results

### 3.1 Demographic and contextual characteristics

A total of 1,300 questionnaires were distributed in this study, and 1,095 valid questionnaires were recovered, with an effective response rate of 84.3%. The average age of the 1,095 nursing students was (21.4 ± 1.08) years, and the mean duration of their internship was (7.82 ± 1.65) months. Details are shown in Table 1.

**Table 1:** Characteristics of nursing interns.

Characteristics	Male	Female	Overall	*p*
	(*N* = 144)	(*N* = 951)	(*N* = 1,095)	
Age				0.013
Mean (SD)	21.6 (1.04)	21.4 (1.08)	21.4 (1.07)	
Hospital level, *n* (%)				0.317
Grade III-A hospital	105 (72.9%)	735 (77.3%)	840 (76.7%)	
Grade II-A hospital	32 (22.2%)	189 (19.9%)	221 (20.2%)	
Other	7 (4.9%)	27 (2.8%)	34 (3.1%)	
Educational level, *n* (%)				0.630
Secondary vocational school	1 (0.7%)	9 (0.9%)	10 (0.9%)	
Junior college	67 (46.5%)	480 (50.5%)	547 (50.0%)	
Undergraduate degree	76 (52.8%)	462 (48.6%)	538 (49.1%)	
Only-child status, *n* (%)				<0.001
No	70 (48.6%)	793 (83.4%)	863 (78.8%)	
Yes	74 (51.4%)	158 (16.6%)	232 (21.2%)	
Family residence, *n* (%)				0.198
Rural	86 (59.7%)	624 (65.6%)	710 (64.8%)	
Urban	58 (40.3%)	327 (34.4%)	385 (35.2%)	
Served as a class cadre, n (%)				<0.001
No	83 (57.6%)	715 (75.2%)	798 (72.9%)	
Yes	61 (42.4%)	236 (24.8%)	297 (27.1%)	
Received scholarships, *n* (%)				0.142
No	105 (72.9%)	631 (66.4%)	736 (67.2%)	
Yes	39 (27.1%)	320 (33.6%)	359 (32.8%)	
Physical activity, *n* (%)				0.001
0	55 (38.2%)	353 (37.1%)	408 (37.3%)	
1	54 (37.5%)	277 (29.1%)	331 (30.2%)	
2–3	23 (16.0%)	283 (29.8%)	306 (27.9%)	
>3	12 (8.3%)	38 (4.0%)	50 (4.6%)	
School position status, *n* (%)				0.002
No	78 (54.2%)	642 (67.5%)	720 (65.8%)	
Yes	66 (45.8%)	309 (32.5%)	375 (34.2%)	
Clinical internship period (month)				0.458
Mean (SD)	7.91 (0.939)	7.80 (1.73)	7.82 (1.65)	

n = number; SD = standard deviation; WPVB = workplace psychologically violent behaviors.

### 3.2 Gender differences in workplace psychological violence (WPVB) scores and their correlation with professional commitment

In this study, the overall incidence of WPVB among nursing interns was 40.0%, with a significantly higher rate observed in female nursing interns (41.4%) compared with their male counterparts (30.6%). In this study, there was a significant difference in exposure to workplace psychological violence (WPVB) between male and female nursing interns (*p* = 0.013). The total scores for WPVB and professional commitment were negatively correlated (r = −0.18, *p* < 0.05). Details are shown in Tables 2–4.

**Table 2:** Indidence rate of WPVB (*N* = 1,095).

Variable	Overall	Male	Female	X^2^	*p*
WPVB				6.162	0.013
No	657 (60.0%)	100 (69.4%)	557 (58.6%)		
Yes	438 (40.0%)	44 (30.6%)	394 (41.4%)		
Individual’ s isolation from work				8.928	0.003
No	635 (58.0%)	100 (69.4%)	535 (56.3%)		
Yes	460 (42.0%)	44 (30.6%)	416 (43.7%)		
Attack on professional status					
No	705 (64.4%)	100 (69.4%)	605 (63.6%)	1.852	0.174
Yes	390 (35.6%)	44 (30.6%)	346 (36.4%)		
Attack on personality					
No	930 (84.9%)	118 (81.9%)	812 (85.4%)	1.156	0.282
Yes	165 (15.1%)	26 (18.1%)	139 (14.6%)		
Direct negative behaviors				1.345	0.246
No	967 (88.3%)	123 (85.4%)	844 (88.7%)		
Yes	128 (11.7%)	21 (14.6%)	107 (11.3%)		

**Table 3:** Scores on dimensions of WPVB and professional commitment.

Characteristics	Male Mean (SD)	Female Mean (SD)	*p*
Professional commitment	2.82 (0.71)	2.72 (0.70)	0.125
Professional effort willingness	2.83 (0.81)	2.70 (0.79)	0.053
Professional career involvement	2.75 (0.85)	2.59 (0.82)	0.029
Positive professional evaluation	2.76 (0.61)	2.89 (0.57)	0.011
Professional value identification	2.97 (0.78)	2.90 (0.77)	0.336

**Table 4:** Correlation between professional commitment and WPVB.

Characteristics	Individual’s isolation from work	Attack on professional status	Attack on personality	Direct negative behaviors	WPVB
Professional effort willingness	−0.17^**^	−0.21^**^	−0.09^**^	−0.07^**^	−0.16^**^
Professional career involvement	−0.14^**^	−0.2^**^	−0.05	−0.03	−0.14^**^
Positive professional evaluation	−0.22^**^	−0.26^**^	−0.18^**^	−0.18^**^	−0.24^**^
Professional value identification	−0.15^**^	−0.17^**^	−0.1^**^	−0.1^**^	−0.15^**^
Professional commitment	−0.18^**^	−0.23^**^	−0.1^**^	−0.18^**^	−0.18^**^

^**^p < 0.05.

### 3.3 Gender differences in the relationship between workplace psychological violence and professional commitment

The hierarchical multiple linear regression was analyzed using the method of gradually including covariates. Model 1 was the unadjusted model; Model 2 adjusted for gender and age; Model 3 further adjusted for hospital grade, educational level, whether being an only child, family residence, whether being a student cadre, whether receiving scholarships, physical exercise situation, whether holding a position at school, and duration of clinical internship. The results showed that in the fully adjusted model (Model 3), female nursing interns had a significantly negative correlation with professional commitment (B = −0.210, SE = 0.030, t = −7.12, *p* < 0.001), and professional status aggression (B = −0.195, t = −8.53) and job isolation (B = −0.169, t = −6.89) were the main influencing dimensions. In the male group, no significant association was observed between psychological violence and professional commitment (Table 5).

**Table 5:** Association between WPVB and professional commitment.

Variables	Model 1					Model 2					Model 3				
	B	SE	t	*p*-value	95% CI	B	SE	t	*p*-value	95% CI	B	SE	t	*p*-value	95% CI
WPVB	−0.141	0.023	−6.00	<0.001	−0.187, −0.095	−0.137	0.024	−5.80	<0.001	−0.183, −0.091	−0.201	0.027	−7.45	<0.001	−0.255, −0.148
Individual’ s isolation from work	−0.124	0.020	−6.06	<0.001	−0.164, −0.084	−0.120	0.021	−5.85	<0.001	−0.160, −0.080	−0.166	0.023	−7.26	<0.001	−0.211, −0.121
Attack on professional status	−0.148	0.019	−7.68	<0.001	−0.186, −0.110	−0.145	0.019	−7.50	<0.001	−0.184, −0.107	−0.187	0.021	−8.83	<0.001	−0.229, −0.146
Attack on personality	−0.084	0.026	−3.26	0.001	−0.134, −0.033	−0.080	0.026	−3.09	0.002	−0.130, −0.029	−0.132	0.030	−4.40	<0.001	−0.192, −0.073
Direct negative behaviors	−0.075	0.027	−2.82	0.005	−0.128, −0.023	−0.071	0.027	−2.65	0.008	−0.124, −0.019	−0.120	0.032	−3.79	<0.001	−0.181, −0.058
Male															
WPVB	−0.120	0.062	−1.94	0.055	−0.243, 0.002	−0.120	0.062	−1.93	0.056	−0.244, 0.003	−0.121	0.077	−1.58	0.116	−0.272, 0.031
Individual’ s isolation from work	−0.120	0.057	−2.10	0.037	−0.233, −0.007	−0.120	0.057	−2.09	0.038	−0.234, −0.007	−0.126	0.070	−1.80	0.074	−0.264, 0.012
Attack on professional status	−0.109	0.053	−2.07	0.0401	−0.214, −0.005	−0.109	0.053	−2.05	0.042	−0.214, −0.004	−0.090	0.064	−1.40	0.164	−0.216, 0.037
Attack on personality	−0.090	0.066	−1.36	0.176	−0.222, 0.041	−0.091	0.067	−1.36	0.176	−0.222, 0.041	−0.095	0.080	−1.20	0.234	−0.252, 0.062
Direct negative behaviors	−0.095	0.068	−1.39	0.166	−0.230, 0.040	−0.096	0.069	−1.40	0.164	−0.231, 0.040	−0.103	0.081	−1.28	0.203	−0.263, 0.056
Female															
WPVB	−0.142	0.025	−5.61	<0.001	−0.192, −0.092	−0.139	0.026	−5.43	<0.001	−0.189, −0.089	−0.210	0.030	−7.12	<0.001	−0.268, −0.152
Individual’ s isolation from work	−0.122	0.022	−5.57	<0.001	−0.165, −0.079	−0.120	0.022	−5.43	<0.001	−0.163, −0.076	−0.169	0.025	−6.89	<0.001	−0.217, −0.121
Attack on professional status	−0.153	0.021	−7.38	<0.001	−0.194, −0.112	−0.151	0.021	−7.22	<0.001	−0.192, −0.110	−0.195	0.023	−8.53	<0.001	−0.240, −0.150
Attack on personality	−0.082	0.028	−2.93	0.003	−0.136, −0.027	−0.077	0.028	−2.74	0.006	−0.132, −0.022	−0.134	0.033	−4.05	<0.001	−0.200, −0.069
Direct negative behaviors	−0.071	0.029	−2.43	0.015	−0.128, −0.014	−0.066	0.029	−2.25	0.025	−0.123, −0.008	−0.117	0.035	−3.34	<0.001	−0.185, −0.048

### 3.4 Mixed linear regression model incorporating WPVB × gender interaction and covariates

The results of the mixed linear regression model including WPVB × gender interaction term and demographic sociological covariates showed that WPVB always had a significant negative main effect on career commitment and the effect was enhanced in the full covariate model. However, the WPVB × Gender interaction term had no statistical significance in all stepwise regression models (Model 1: Estimate = −0.022, *p* = 0.7424; Model 2: Estimate = −0.019, *p* = 0.7742; Model 3: Estimate = −0.040, *p* = 0.53898), indicating that gender did not significantly moderate the relationship between WPVB and professional commitment.

## 4 Discussion

This study examined gender disparities in the incidence of workplace psychological violence among nursing interns in China, utilizing survey data from 1,095 participants across 14 medical colleges in Shandong Province. The results indicated an overall prevalence rate of psychological violence at 40.0%. Notably, female interns experienced a higher rate (41.4%) compared to their male counterparts (30.6%). This result aligns closely with the research by Cai et al. (24), who surveyed 526 intern nurses from six institutions in Hubei, Guangdong, and Gansu provinces, reinforcing the pervasive nature of psychological violence among intern nurses and the consistent regional trend indicating females face greater risk. These gender differences may be attributed to societal gender role stereotypes. Such stereotypes represent deeply ingrained perceptions within a cultural context about the behavioral and personality traits deemed appropriate for each gender (32). Historically, influenced by traditional views, nursing has been perceived as a “feminine” profession, with women believed to exhibit more care, patience, and empathy, thus better fulfilling societal expectations of the nursing role (33). Against this background, patients and their families often have higher professional expectations for female nursing interns (34). However, such high expectations are often accompanied by negative emotions such as disappointment and dissatisfaction (2). When female nursing interns fail to meet the expectations due to insufficient clinical experience and unskilled operation, they are often attributed to “not meeting gender role expectations” and subjected to psychological violence such as verbal abuse, attitudinal rejection, and social isolation. This ultimately leads to a higher rate of exposure to psychological violence among female nursing students. This result is different from Albashtawy’s (35) and Wang Chao’s (36) findings on employed nurses that male employed nurses reported a higher level of exposure to psychological violence. The difference may be related to the career development stage of the research subjects. Employed nurses undertake more independent clinical responsibilities, face greater work pressure and professional expectations, and are more likely to become targets of conflict or suffer targeted violence in high-intensity environments (36). At the same time, in most Asian countries, the gender stereotype of nursing occupation is more prominent, and male nurses are often faced with implicit questioning and professional identity pressure due to gender bias, making them more likely to become targets of psychological violence (37). In conclusion, gender differences in workplace psychological violence are not static but rather exhibit dynamic characteristics that vary with the stage of career development. During the internship phase, women are more susceptible to psychological violence, whereas men may face a higher risk during the working phase. This indicates that future intervention strategies for psychological violence should fully account for the distinct needs arising from both career stage and gender, implementing stratified, categorized prevention and targeted support measures.

The present study found that the overall psychological violence experienced by nursing interns was negatively correlated with their professional commitment level, which is consistent with François Courcy’s (38) findings in a survey of 1,228 university employees. In addition, many researchers who focused on nurses and explored the relationship between psychological violence and professional commitment also reached the same conclusion (39, 40), indicating that workplace psychological violence has a negative impact on professional commitment. According to Conservation of Resources Theory (21), workplace psychological violence essentially involves the dual consumption of internal and external resources for nursing trainees: On the one hand, the anxiety and depression caused by violent incidents will consume individuals’ psychological resources and weaken their inherent resilience in coping with occupational stress (41). On the other hand, interpersonal trust breakdown and professional dignity damage caused by violence would further deprive nursing trainees of social support resources obtained from clinical settings (42). When the rate of resource depletion surpasses replenishment, trainee nurses may safeguard their resources by diminishing professional commitment and weakening their professional identity. This can lead to a decline in their overall professional dedication. Consequently, it is imperative for nursing education and management departments to institute a comprehensive intervention system addressing psychological violence faced by trainee nurses. First, during the internship phase, structured training on identifying and responding to psychological violence should be provided. This will equip trainees with robust psychological defense strategies, mitigating the toll of violent incidents on their personal resources. Second, enhancements to the reporting and intervention mechanisms related to psychological violence in the internship setting are essential. Aiming for a zero-tolerance environment offers external support, aiding trainee nurses in preserving their resources. This not only curtails psychological violence but also stabilizes their professional identity and commitment. Lastly, consistent evaluation and refinement of these interventions are crucial. Managers must ensure their efficacy and offer ongoing mental health assistance to trainee nurses.

This study mainly explored the moderating effect of gender on the relationship between WPVB and professional commitment among nursing interns. Although the results of stratified multiple linear regression analysis showed that the association between workplace psychological violence and professional commitment was statistically significant only in female nursing interns, while no significant association was found in male group; however, the results of formal test including interaction terms indicated that gender did not significantly moderate the relationship between these two variables. That is to say, this study failed to confirm that gender was a significant moderator variable for the relationship between WPVB and professional commitment among nursing interns. The conclusion of this study is opposite to Hengxi Chen et al. (2). The reasons are speculated to be related to the following aspects:

The clinical internship is a crucial stage in nursing education, serving not only as a key transitional period from theoretical learning to clinical practice for nursing interns but also as a core opportunity for the transformation of their professional identity from theoretical cognition to practical perception (43). During this period, the professional commitment of nursing interns is still being shaped by the external environment, and its level is primarily influenced by factors such as the clinical internship experience, the quality of teaching guidance, and the mastery of basic clinical skills. The corresponding professional identity constitutes only a preliminary perception and tentative recognition of the nursing profession and has not yet formed a stable individual self-value system (1, 12). Gender, as a deep-seated social psychological variable formed through long-term socialization, exerts an indirect, gradual, and profound impact on professional psychology, it must interact with a mature professional identity and stable professional role cognition to alter the pathway through which external stressors affect professional psychology (44, 45).

In the context of nursing interns whose professional commitment has not yet been fully established, gender cannot significantly modify the relationship between WPVB and professional commitment. Its influence can only be manifested through group differences; therefore, it does not achieve a statistically significant moderating effect. Moreover, compared with full-time nurses, the professional role positioning of nursing interns and the exposure characteristics of WPVB may further weaken the moderating effect of gender. Full-time nurses, in contrast to nursing interns, undertake formal nursing responsibilities and have formed stable professional role cognition and behavioral patterns, their professional commitment is a mature psychological structure formed through long-term clinical practice, and their responses to various stressors in the professional environment are more individualized and have deeper psychological correlations (46); and the WPVB encountered by full-time nurses are usually deep, continuous and targeted attacks on their professional identity, such as possible career ability doubt leading to denial of career status, workplace ostracism caused by job competition, persistent verbal violence triggered by escalating nurse–patient conflicts, etc. (47). Such high-intensity stressors can fully activate the deep psychological defense mechanism of individuals, so that gender-induced perceptual differences and coping patterns are fully manifested, it will change the strength of WPVB’s effect on professional commitment, and ultimately produce a significant regulatory effect (48, 49). As nursing interns belong to novice groups in clinical settings, their professional role is defined as “learners and practitioners”. They have not yet participated in formal work tasks and career competition. The WPVB they encounter are mostly mild and general interpersonal negative behaviors related to lack of clinical skills, such as occasional verbal criticism caused by unskilled operation, brief negation caused by lack of communication experience, temporary exclusion during team integration process, etc. (34). These low-intensity and non-targeted stressors can only trigger apprentice nurses’ superficial emotional reactions and behavioral adjustment, but cannot penetrate into individual’s deep psychological structure. Therefore, gender has not played a regulatory role in the relationship between its professional commitment and psychological violence.

Gender did not play a role in the relationship between psychological violence and professional commitment among nursing interns, this may be related to the inherent characteristics of the nursing profession. The nursing profession in China is dominated by female groups, and the characteristic of a female-led industry further strengthens the deep binding of the nursing profession with the gender of women, resulting in highly fixed gender expectations for the nursing profession in society. These expectations then permeate into the clinical practice environment and become an important constraint on the formation and stress response of professional commitment among male and female nursing students (34). In social cognition, “being careful, patient, good at empathy, and skilled in interpersonal communication” are classified as feminine traits (32), long-term integration with the core competencies of the nursing profession has made the process of forming professional commitment among female nursing students align with industry expectations, and their identification with and commitment to the nursing profession more of a positive response to the gender expectations of the industry; whereas male nursing students choose the nursing profession and form professional commitment mostly due to rational decisions such as individual career planning, employment choices, and personal ability matching (33, 43). Although this seems to differ from the gender expectations of the industry, the maintenance and development of their professional commitment still needs to be achieved within the existing ecological framework of the nursing profession and cannot be separated from the core essence of the profession. Whether it is the natural integration of female nursing interns into industry expectations or the rational adaptation of male nursing intern, their professional commitment is regulated and constrained by the overall ecology of the nursing profession (2, 50). As a typical negative factor in nursing industry, WPVB is generated from the inside of nursing industry and its generation, dissemination and action path are determined by the nursing industry itself. The negative impact on professional commitment of nursing interns is more due to the transmission of negative factors within the nursing industry than dominated by individual gender traits (51). Therefore, gender as an individual-level variable cannot break through the overall constraints of the ecosystem of the nursing industry, and it is also difficult to change the core role relationship between WPVB and professional commitment. Thus, gender does not play a moderating effect. In addition, from the perspective of group size, female groups absolutely dominate the nursing industry, making them the mainstream group of the industry ecosystem. While male groups, as a minority group (52), their behavioral and perceptual differences brought about by gender characteristics will be further weakened under the framework of inherent attributes of the nursing industry. In the clinical practice environment, the WPVB encountered by nursing interns are mostly non-gender-specific negative behaviors based on common problems in the industry, rather than differentiated attacks targeting specific genders. This mode of action of stressor without distinction further reduces the possibility of gender playing a moderating effect; at the same time, male nursing interns belong to the minority group in the nursing industry, and it is difficult to change the existing ecological system model of the industry with their own gender characteristics, they can only actively adapt to this female-dominated professional environment. The potential influence of their gender characteristics on the relationship between WPVB and professional commitment may be gradually eliminated by the industrial environment in the process of adaptation, therefore, gender has no significant moderating effect.

Gender did not play a moderating role in the relationship between psychological violence and professional commitment among male and female nursing interns, this may be related to differences in coping patterns between male and female nursing students. When faced with this occupational stressor of psychological violence, male nursing interns tended to adopt problem-focused coping strategies, preferring to reduce the impact of stress by actively communicating, filling capacity gaps, and seeking external support (23), while female nursing interns were more likely to adopt emotion-focused coping strategies, often using passive avoidance, emotional internalization, and passive acceptance to deal with negative experiences (53–55). The positive coping of males only delayed the rate of resource depletion, preventing it from rapidly reaching the critical threshold that triggers a decline in professional commitment, the negative coping of females accelerated the process of resource depletion, allowing them to cross the threshold more quickly and trigger a decrease in professional commitment. This difference only changed the timing and degree of manifestation at which psychological violence affected professional commitment, it did not fundamentally change the intensity of the negative effect or the core path through which psychological violence affects professional commitment. Therefore, there was no gender moderation effect. Psychological capital, as a key mediating variable connecting psychological violence and professional commitment, its consistency across genders in terms of mechanism of action further confirmed this conclusion. Psychological capital is the core internal resource for nursing interns to cope with occupational stress and maintain professional commitment. The negative impact of psychological violence on professional commitment was mostly achieved through the depletion of nursing students’ psychological capital (24), In this study, gender differences in coping patterns were only reflected in the degree of psychological capital depletion: female nursing students’ negative coping strategies led to a more rapid and significant depletion of their psychological capital, whereas male nursing interns positive coping strategies resulted in a slower and less significant depletion. However, gender did not alter the mediating mechanism of psychological capital between psychological violence and professional commitment; it merely influenced the strength of the mediating variables. This superficial difference does not support gender as a significant variable regulating the core relationship between psychological violence and professional commitment, nor does it facilitate the effective formation of the moderating effect.

This study still has certain limitations. First, the cross-sectional design of this study makes it impossible to establish a causal relationship between psychological violence and professional commitment. Subsequent longitudinal follow-up studies can be conducted to dynamically observe the interaction effect between the two over time; secondly, the research sample is all from Shandong Province, which may have regional representation bias. In the future, the sampling scope can be expanded to include intern nurses from medical institutions at different levels and types; thirdly, all data in this study were collected through self-report questionnaires, which may lead to common method bias and social desirability bias. In addition, participants were recruited from multiple medical institutions in Shandong Province, and clustering effects by location were not considered in the statistical analysis, which may affect the accuracy of the research results; fourthly, nursing practitioners in China are predominantly female, and the proportion of male nursing students remains low even in the nursing education stage. This actual industry background has led to a significant gender imbalance in the research sample (144 males and 951 females), which further results in insufficient statistical power for the analysis of the male group in this study. Thus, future verification through large-sample studies with a relatively balanced gender distribution is needed; finally, influenced by traditional Chinese culture, some interns who have experienced workplace violence may be unwilling to report their true situation due to concerns about being labeled or worried that it will affect their internship evaluation (56). Therefore, in the data collection stage of this study, the anonymous filling principle was particularly emphasized to reduce the impact of the above bias on the authenticity of data as much as possible.

## 5 Conclusion

This study undertook a cross-sectional survey of 1,095 intern nursing students from 14 medical colleges in Shandong Province to systematically investigate the correlation between workplace psychological violence and professional commitment, as well as the moderating influence of gender. The overall incidence rate of psychological violence among nursing interns was 40.0%. Among them, the incidence rate for female interns (41.4%) was significantly higher than that for male interns (30.6%). Workplace psychological violence was significantly negatively correlated with professional commitment. Although stratified analysis only observed a significant negative correlation in the female group, the formal regulatory test that included interaction terms confirmed that gender did not play a significant moderating role in the relationship between psychological violence and professional commitment. This might be related to the fact that the professional identity of nursing students has not yet stabilized, the violence encountered during the internship stage is mostly non-targeted events, and the overall environment of the nursing profession. The results of this study suggest that a universal system for preventing and intervening in psychological violence should be established to improve nursing interns’ ability to recognize and respond to psychological violence, and strengthen the psychological resilience training for all intern nurses, in order to reduce the incidence rate of psychological violence among nursing interns and enhance their professional commitment.

## Data Availability

The datasets presented in this article are not readily available because the data that support the findings of this study are available on request from the corresponding author. Requests to access the datasets should be directed to JingFang Zhao, jingfangzhao@126.com.
